# Triple assessment breast clinics: The value of clinical core biopsies

**DOI:** 10.1007/s11845-023-03445-z

**Published:** 2023-08-08

**Authors:** Rahmani Maha, Johnston Alison, Sugrue Michael, Varzgalis Manvydas

**Affiliations:** 1 Donegal Clinical Research Academy, Letterkenny University Hospital, Letterkenny, Co. Donegal Ireland; 2 Department of Breast Surgery, Letterkenny University Hospital, Letterkenny, Co. Donegal Ireland; 3 University College Dublin, Dublin, Ireland; 4 University Of Galway, Galway, Ireland

**Keywords:** Breast cancer diagnosis, Clinical core biopsies, Percutaneous free-hand core biopsy, Quality improvement, Resource management, Symptomatic breast units, Triple assessment

## Abstract

**Background:**

Triple Assessment Breast Clinics are designed for rapid diagnosis of symptomatic patients. When there is no concordance between clinical and radiological assessment, clinicians perform clinical core biopsies. In patients with a clinically suspicious examination (S4, S5) and normal imaging, clinically guided core biopsy should be performed as per NCCP guidelines. However, substantial research does not exist on the diagnostic value or use of clinical core biopsies in non-suspicious palpable (S3) lesions and practices differ in each health system.

**Aims:**

The aim of this research was to assess the diagnostic value of clinical core biopsies in nonsuspicious, probably benign palpable breast lesions (S3) where image guided cores were not indicated (R1/R2).

**Methods:**

The cohort consisted of patients undergoing clinical core biopsies at a Symptomatic Breast Unit from January 2014 to 2019. Data regarding patient demographics, outcome of triple-assessment and incidence of malignancy were obtained from a prospectively maintained database and results were analysed using Minitab 2018.

**Results:**

Three hundred and sixty patients had a clinical core biopsy performed in this period. Clinical examination scores for these patients were S1/S2 (66), S3 (277), S4 (15), and S5 (2). Radiology Scores were R1/R2 (355) and R3(5). Two patients with clinical score S3 (0.6%) were diagnosed with breast cancer due to their clinical cores. Both patients had normal mass imaging. There was no association between uncertain palpable breast lesions (S3), and atypia or malignancy on biopsy results when breast imaging was normal (*P* = 0.43, χ^2^ test).

**Conclusion:**

Despite clinical core biopsies being used in triple assessment, there is no certainty in their value except that there is high clinical suspicion. Imaging modalities are constantly improving and are already well established. When the patient is assigned a clinical score of S3 and has normal radiology, a clinical core biopsy is not required in most cases.

## Introduction

Breast cancer diagnosis and management in Ireland has changed significantly over the past two decades. Centralisation of breast services has resulted in 9 national symptomatic breast units (SBUs), one of which is the Breast Centre Northwest (BCNW), a satellite centre, which employs a triple assessment approach to provide a rapid diagnostic service [1–4]. Triple assessment consists of a detailed personal and family history, a clinical breast examination (CBE), and radiological investigations which include obtaining image guided core biopsies if required [5]. A standardised universal grading system is used for clinical, radiological, and pathological scoring of each breast according to the most suspicious lesion (Table 1) [6–8].

**Table 1:** Classifications

Clinical exam	Radiology	Biopsy pathology
S1 — Normal	R1 — Negative	B1 — Inadequate sample
S2 — Benign	R2 — Normal/benign	B2 — Normal/benign
S3 — Indeterminate, probably benign	R3 — Indeterminate/probably benign	B3 — Uncertain
S4 — Suspicious, probably malignant	R4 — Suspicious	B4 — Suspicious
S5 — Malignant	R5 — Highly Suspicious	B5a — Malignant (non-invasive) B5b — Malignant (invasive)

If there is no concordance between clinical and radiological assessment, a clinical core biopsy is performed by the investigating clinician or a more conclusive answer. In patients with a clinically suspicious examination (S4, S5) and normal imaging (mammography and ultrasound), clinically guided core biopsy should be performed as per NCCP guidelines [9]. Clinical core biopsies remain a valuable tool in the diagnosis of breast cancer particularly in the management of clinically suspicious radiographically occult malignancies [10]. However, substantial research does not exist on the diagnostic value or use of clinical core biopsies in non-suspicious palpable (S3) lesions and practices differ in each health system. The routine use of clinical core biopsies requires extra clinical measures, lab resources, and specialist time and therefore needs to be evaluated.

The aim of this research is to objectively evaluate the results of the clinical cores obtained in BCNW and to assess the diagnostic value of clinical core biopsies in nonsuspicious, probably benign palpable breast lesions (S3) where image guided cores were not indicated (R1/R2).

## Methods

### Study design and population

An ethically approved retrospective review of all patients who underwent triple assessment with a clinical core biopsy in BCNW from January 2014 to April 2019 was conducted. All clinically guided core biopsies were performed on patients using a free hand Medax 14G * 100 mm CAESAR soft tissue fully automatic spring-loaded biopsy system, Ref: CS14100-00. Data was collected from Dendrite Clinical Systems Ltd,^1^ a prospectively maintained internationally recognised clinical registry.

Demographic data and clinical, radiological, and biopsy-pathology scores were collected. Clinical and radiological findings were correlated to pathological findings. The level of association between clinical scores and biopsy results was assessed and the diagnostic yield of clinical core biopsy evaluated.

Patients were included in this study if they met the following criteria: a full triple assessment performed on the initial date of attendance, underwent a clinical core biopsy, and had full clinical, radiological, and pathology scores documented. Patients were not included if they had clinically suspicious lesions including those that as suspicious for Paget’s disease. Patients were not included if they underwent a punch, stereotactic, or image guided biopsy or if their clinical condition, such as dementia, resulted in incomplete assessment.

### Statistical analysis

Statistical analysis was conducted using Minitab 2018. Mean, standard deviation (SD), median, and interquartile range were calculated for continuous and count data. Chi-square test was used as appropriate for categorical and continuous variables to evaluate level of association. Data was considered statistically different if *p*-value was < 0.05*.*

## Results

### Patient variables

Three hundred fifty-two patients (348 females and 4 males) underwent core biopsies over the 51-month period. Mean age was 52 (24–94); median 59; modal 39; SD 13.3.

### Triple assessment results

**Graph 1:**
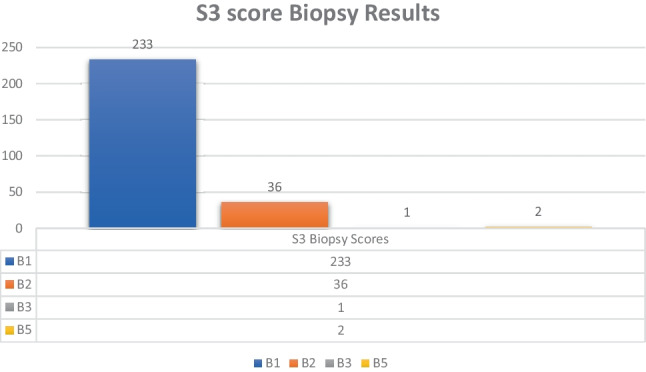
S3 score biopsy results

Detailed results of triple assessment are presented in Table 2. 349/352 (99%) received a final benign diagnosis of B1 or B2. 2/352 (0.6%) were diagnosed with malignant disease: one with a B5a, non-invasive diagnosis and the other with a B5b score for invasive disease. 1/352 (0.3%) received a final score of B3 (Graph 1).

**Table 2:** Triple assessment scores

Mode of assessment	*N* (%)
Clinical score total	352
S1	6 (1.7)
S2	59 (16.8)
S3	271 (76.9)
S4	14 (4.0)
S5	2 (0.6)
Radiology score total	352
R1	113 (32.1)
R2	236 (67.0)
R3	3 (0.9)
Pathology score total	352
B1	301 (85.5)
B2	48 (13.6)
B3	1 (0.3)
B5	2 (0.6)

Both patients who were diagnosed with breast cancer (B5 on biopsy) had occult findings on mammogram and ultrasound. The patients were given clinical scores of S3 and were both aged over 70. One of these patients had a palpable DCIS. She was given a score of R3 due to the presence of focal skin oedema and diffuse skin thickening. There was no mass abnormality detected, and the lump was mammographically occult. It was an agreement between the radiologist and surgeon that the surgeon perform a clinical core biopsy for this patient. The other patient was given a score of R2 for dense breast tissue. There was no abnormality detected of the palpable lump. She was subsequently diagnosed with retro- areolar infiltrating ductal carcinoma which is difficult to see on imaging.

### Chi-square test for association

The chi-square test of association was performed to assess the diagnostic yield of clinical core biopsies taken in non-suspicious, probably benign, palpable breast lesions (S3) where image-guided cores were not indicated. There was no association between uncertain palpable breast lesions (S3) and atypia or malignancy on biopsy results when breast imaging was normal (*p* = 0.43). Table 3.

**Table 3:** Calculations for chi-square test for association

	Expected	Observed
Indeterminate clinical score with normal/benign biopsy results	272	274
Indeterminate clinical score with uncertain biopsy results	2	1
Indeterminate clinical score with malignant biopsy results	3	2

## Discussion

Breast cancer is the second most common cancer in women in Ireland with more than 3500 cases being diagnosed per year [10, 11]. For this reason, each patient that is referred to a SBU is treated as having a potential cancer diagnosis and the surgeon must aim to disprove the presence of cancer.

Image-guided biopsy has become an established technique for obtaining a diagnosis of breast cancer; however, both palpation-guided and image-guided biopsies are routinely used for histopathological sampling of palpable breast masses [12]. To minimise the number of cancers that are missed, clinical core biopsies are performed when clinical and radiological scores are not concordant [13].

In our observational study for a single urban unit, only 2/352 (0.6%) patients were diagnosed with malignancy after receiving clinical core biopsies.

The aim of triple assessment is to provide a rapid outpatient approach to diagnosis and allow for the early intervention in the treatment of breast cancer [5]. It is therefore necessary that all aspects of the triple assessment process are specific and sensitive so accurate results can be delivered to patients and the SBUs can serve their intended purpose.

### Sensitivity of clinical breast examination

The CBE is performed by the breast surgeon prior to any imaging and/or biopsy. It consists of a visual inspection and palpation of the breasts and lymph nodes. Both breasts should be assessed for any lumps, nodules, or skin differences such as dimpling, skin retraction, or discolouration. The size, side, and specific locations of each lump are noted, and a clinical score is given as per national guidelines.

Historically, a considerable number of breast cancers were detected by CBE alone, but today, it is less clear how important CBE is in the early detection of breast cancer as women receive regular screening with high quality mammogram. CBE may contribute to earlier detection of breast cancer in women under the age of forty for whom mammography is not recommended. At present, no standardised system exists for interpreting and reporting the results of CBE [14].

The National Breast and Cervical Cancer Early Detection Program published values for CBE sensitivity (58.8%) and specificity (93.4%) [15]. Another study found a range for sensitivity from 40 to 69% and a specificity of higher than 93% [16].

Recent estimates suggest that screening CBE sensitivity depends on several factors including the force of palpation, search technique applied, examination time, and level of experience of the examining physician [17].

While emphasis often is placed on achieving high sensitivity, achieving high CBE specificity is important in minimising the risk of false-positive results and the consequent unnecessary medical procedures and stress for patients [14].

The way in which a CBE is performed varies significantly from person to person. It depends on patient characteristics and examiner characteristics. Patient characteristics include breast density and nodularity. It can be difficult to determine if a mass is present by physical examination in more dense breast tissue such as with premenopausal women. All breasts also have variable combinations of glandular tissue, fibrosis, and fat which can make it difficult to assess a true mass. True masses are generally asymmetrical in relation to the other breast, distinct from the surrounding tissues, and 3-D. Cysts cannot reliably be distinguished from solid breast masses by palpation [18].

Women presenting with a palpable breast lump should undergo a thorough CBE as well as imaging evaluation as not all breast masses will exhibit distinctive physical findings. Recommended imaging options in the context of a palpable mass include diagnostic mammography and targeted-breast ultrasound and are dependent on patient age and degree of radiologic suspicion [18].

### Sensitivity of radiological examination — mammogram and ultrasound

Patients will receive mammograms and/or ultrasounds as part of their triple assessment appointment if certain criteria, such as age and presence of a focal clinical or radiological abnormality, are met. The findings of each mammographic, and ultrasonography examination are classified according to The Royal College of Radiologists Breast Group breast imaging classification [19].

Mammograms are widely used as a screening tool for breast cancer but as with every diagnostic test they have their limitations [20]. Interpretation of mammograms in women under the age of forty is challenging due to the typically denser breast parenchyma when compared to older women [21]. For this reason, ultrasonography is the primary imaging modality used for women under 40 years of age [22, 23].

Ultrasound is preferably targeted specifically to the palpable finding. A major advantage of ultrasound is the ability to directly correlate the clinical and imaging findings. Many palpable masses can be characterised as benign using ultrasound. These may include simple cysts, clustered microcysts, or sebaceous cysts [24]. A recent meta-analysis of ultrasound for breast cancer detection globally found that ultrasound had an overall pooled sensitivity and specificity of 80.1% (95% CI, 72.2 to 86.3%) and 88.4% (95% CI, 79.8 to 93.6%), respectively [25]. Ultrasound is the modality of choice in pregnant and lactating women because tissue density limits mammographic evaluation.

There is variation in the sensitivity and specificity rates for the modalities due to different study populations and reporting protocols; however, reported sensitivities for mammography range between 57 and 97% and for ultrasound between 49 and 100%. Specificity for mammography ranges between 36 and 97% and for ultrasound between 29 and 100% [21, 26–29].

It has been shown that sensitivity improves with age and a combination of clinical exam, mammogram, and ultrasound has additional benefits of a higher sensitivity and specificity rate [30]. Ultrasound has high sensitivity (95.7%) and high negative predictive value (99.9%) in women 30–39 years of age with focal signs or symptoms [23].

### The value of clinical core biopsies

Based on our research we have found that although clinical core biopsies are being used for non-suspicious palpable breast lumps, there is no certainty in their value. Imaging modalities are constantly improving and are already well established with more objective results than a CBE. Many risk factors such as age, breast density, and family history must be considered before planning a core biopsy.

Core needle biopsy requires patient preparation such as cessation of antithrombotic medication. Local anaesthesia is used to make a small skin incision, and complications, although rare, can still happen. These include haematoma, pneumothorax, vasovagal symptoms, infection, and seeding of malignant tumour cells [31].

Clinical core biopsy is associated with higher false negative rates due to missed biopsies. The appropriate area of the lesion must be removed for correct histological interpretation; there is a possibility that the lesion is left behind and remains in the breast. Clinical core biopsy has not been associated with being less costly or less time-consuming; therefore, they should only be used when clinical suspicion is extremely high [32].

Clinical core biopsy has been found to have a sensitivity of 91% (0.8–0.96) and specificity of 98% (0.95–1.00) as well as a false negative rate of 13% [33].

Our study found that clinical core biopsies did not add any diagnostic value in S3 lesions that had normal radiology. In the cases where it is possible the patient should be called for reassessment, a timely manner before a clinical core biopsy is performed so that more than one clinician can assess the lump to ensure concordance in scores.

There is no association between S3 lumps and atypia or malignancy when patients had a clinical core biopsy performed.

### Limitations to study

This was a study of a single urban centre and may not reflect the practices in other centres. We also need to do a further study on the follow-up of patients who had a clinical core biopsy and whether they were diagnosed with breast cancer later in life.

## Conclusion

In conclusion, in our experience, a clinical core biopsy did not add any value when patients were assigned a clinical score of S3 with normal radiology. However, clinical core biopsies are still recommended when the clinician’s degree of suspicion is high enough to warrant a score S4/S5.

There is no significant association between S3 lesions and atypia or malignancy. We should avoid taking a clinical core biopsy in every S3 lesion with normal radiology; however, these patients should be reassessed in a timely manner or by a second clinician to avoid missing a malignancy. Further guidelines are necessary to improve breast cancer care in Ireland.

## Footnotes

1 Dendrite Clinical Systems Ltd enables clinicians to create and manage their own databases to collect, analyse and report local data — for clinical audit, research, and benchmarking to track and improve clinical practice and outcomes.
